# Utility of performance-based outcome measures (PBOMs) used in fall risk assessment tools for older adults

**DOI:** 10.1016/j.dialog.2022.100043

**Published:** 2022-09-15

**Authors:** Kevin M. Parcetich, Daniel G. Miner, Arco Paul, Lane Wildman

**Affiliations:** Department of Physical Therapy, Radford University Carilion, 101 Elm Ave., 8^th^ Floor, Roanoke, VA 24013, United States of America

**Keywords:** Older-adult, Fall risk assessment, Diagnostic accuracy

## Abstract

This study investigated the diagnostic accuracy of different clusters of performance based outcome measures (PBOMs) recommended by two consensus-based guidelines: Stopping Elderly Accidents, Deaths, and Injuries (STEADI), and those recommended by a systematic review completed by the American Physical Therapy Association and Academy of Geriatric Physical Therapy (APTA-SR, APTA-SR3). 33 community-dwelling older adults (25 females, 8 males) aged mean 79.45 ± 7.64 years participated in this study. Participants completed a fall history questionnaire and were evaluated via a battery of PBOMs for comparative analysis. The diagnostic accuracy of each PBOM cluster was analyzed retrospectively (previous 1 year fall history) and prospectively (6 month follow up). Retrospective analysis revealed the APTA-SR3 had the highest clinical utility and diagnostic accuracy: Sp 88.24% (63.56–98.54), Sn 62.5% (35.43–84.8), LR+ 2.35 (1.22–4.53), LR- 0.19 (0.05–0.73), accuracy 70.22% (51.83–84.81). Prospective analysis revealed the cluster of the APTA-SR and APTA-SR3 had identical diagnostic accuracy: Sn 100% (39.76–100), Sp 43.75% (19.75–70.12), LR+ 1.78 (1.15–2.74), LR- 0 (0), accuracy 60.62% (36.63–81.36). The APTA-SR 3 cluster demonstrated the highest diagnostic accuracy and in this study was the most effective and efficient group of PBOMs to identify fall risk in community dwelling older adults.

## 1 Introduction

According to the World Health Organization (WHO), falls are a major global public health problem with an estimated 684,000 fatal falls occurring each year [1]. In all regions of the world, death rates are highest among adults over the age of 60 years old [1]. Estimates suggest that about 1 in 3 older adults living in the community will experience a fall resulting increased morbidity, mortality and health care costs [[2], [3], [4], [5], [6], [7], [8]]. Non-modifiable risk factors such as age, sex, and prior fall history are well established predictors of falls, but provide little insight into the possible body structure/function and movement related impairments that might predict fall risk [[9], [10], [11]]. Modifiable risk factors include strength, joint range of motion, motor control, balance, vision, vestibular system abilities, and general health status, provide insight into possible body structure/function and movement related impairments that might increase fall risk and are important potential targets for therapeutic exercise interventions such as balance training [[12], [13], [14]]. A comprehensive evaluation of the modifiable risk factors associated with fall risk, especially the balance system is essential for the practicing rehabilitation specialist, such as a physical therapist, to identify older adults at risk for falling.

In clinical practice, standardized assessments such as performance-based outcome measures (PBOMs) are used to evaluate fall risk. These assessments help the clinician identify possible impairments within the different components of the balance system that may increase an individual's risk for falls [15]. The balance system is defined as the interaction of biomechanical constraints, stability limits/verticality, anticipatory postural adjustments, postural responses, sensory orientation, and stability in gait [16]. Most PBOMs fail to assess all of the components of the balance system resulting in clinicians often utilizing multiple different PBOM's to increase confidence when interpreting examination findings related to fall risk [17]. Previous research has analyzed the diagnostic accuracy and predictive ability of individual fall risk PBOMs, such as the Timed Up and Go Test (TUG), but there is limited research investigating the diagnostic accuracy and predictive ability when clustering multiple different PBOMs [[18], [19], [20], [21]].

The Center for Disease Control's Stopping Elderly Accidents, Deaths, and Injuries (STEADI) provides a cluster of outcome measures to predict risk of future falls [22]*.* The STEADI was developed using clinical practice guidelines (CPGs) from the American and British Geriatric Society and consists of 3 components: a self-report screening measure, a cluster of PBOMS to identify fall risk, and suggested interventions to reduce fall risk (https://www.cdc.gov/steadi/materials.html). The PBOMs used in the STEADI are the TUG, 30 s Chair Stand Test (30SCST), and the 4-Stage Balance Test (4SBT). The TUG is a measure of a person's fundamental physical function and risk of falling. Participants are told to stand up from a chair with a seat height of 46 cm, walk three meters, turn, and return to sitting as quickly as possible [23]. The 30SCST assesses the number of times in 30 s a person can stand and return to sitting from a 46 centemeter high chair without using upper extremities for assistance [24]. The 4SBT assesses a person's fall risk by having participants stand in four increasingly difficult postures (feet together, modified tandem stance, tandem stance, single limb stance) for at least 10 s without shifting the feet or seeking assistance [25].

A recent systematic review sponsored by the 10.13039/100001943American Physical Therapy Association (APTA) and the Academy of Geriatric Physical Therapy (AGPT) advocated for the use of a different cluster of PBOMs [26]*.* The cluster of PBOMs (APTA-SR) suggested in the review include the Berg Balance Scale (BBS), TUG, Single Limb Stance Time (SLST), 5 Times Sit to Stand (5TSTS), and Self-Selected Walking Speed (SSWS). The BBS is considered the reference standard for assessing balance in older adults [27]. It consists of 14 items designed to measure mobility tasks related to daily activities. Each item receives a score of 0 to 4, where a score of 0 represents an inability to complete all of the items and a score of 4 represents the ability to complete the task independently, yielding a total possible score of 56 [28]. The SLST is a simple test for measuring static aspects of balance that can be used in a variety of settings and requires minimal equipment or training [29]. It is a timed test that requires participants to stand barefoot on one limb with their arms crossed and is completed with eyes open and with eyes closed [29]. The 5TSTS is commonly used to assess lower extremity strength and balance. The test records the time it takes for a participant to stand fully from a 43-cm height chair with their arms crossed on their chest for five consecutive repetitions [30]. The SSWS is a timed test that requires participants to walk at their “usual and comfortable speed” across a 10 m path [31]. This test provides insight into an older adults current and future health function [32,33]. Each outcome measure from the STEADI and the APTA-SR has established validity to assess fall risk in community dwelling older adults [24,25,[34], [35], [36], [37], [38], [39], [40], [41], [42]].

A subset of the proposed cluster (APTA-SR3) consists of the BBS, TUG and 5TSTS was also suggested. The authors of the systematic review advocated for the calculation of the cumulative post-test probability from all PBOM in order to quantify an individual's risk for future falls.

The purpose of this study is to compare the diagnostic accuracy of the clusters of PBOMs used in the STEADI, APTA-SR and APTA-SR3 to learn which set of outcome measures bests evaluates current and future fall risk in community-dwelling older adults.

## 2 Methods

### 2.1 Participants

A convenience sample of 33 healthy older adults were recruited from a local independent living community (range 66–93 years) (Table 1). Signed informed consent was obtained from all participants. The study design was approved by the University Institutional Review Board. Participants were included if they were over the age of 60, without cognitive or neurological disorder, recent injury or comorbidity limiting ambulation or unsupported standing, or any other contraindication to participate in a fall risk assessment as indicated by a physician.

**Table 1:** Participant Characteristics; SD indicates standard deviation.

Characteristic	Participants
Age (mean ± SD)	79.45 ± 7.64 yrs
Sex, female %	75.8
Participants (%) reporting “one fall or more in the past year”	51.5
Participants (%) reporting “feelings of unsteadiness”	60.6
Participants (%) reporting “need for assistive device”	48.5
Participants (%) reporting “impaired sensory condition in feet”	30.3
Participants (%) reporting “impaired vision requiring glasses or contacts”	96.7
Participants (%) reporting “requires hearing aids”	33.3
Participants (%) reporting “currently taking medications causing lightheadedness, dizziness or needed for sleep or mood”	81.8
Participants (%) reporting “depression or depression like symptoms”	24.2
Activities Specific Balance Confidence Score (mean ± SD)	73.75% ± 20.21

### 2.2 Assessment using PBOMs

Participants were contacted via telephone to complete a medical and fall risk screening questionnaire before completing a fall risk assessment. The results from the questionnaire were used to identify fallers and non-fallers within the past year. A fall risk assessment was then performed on all participants on a single day within the following month.

On the day of the fall risk assessment, the older adults completed each of the seven PBOMs used in the STEADI and APTA-SR tools in a randomized order. Following the administration of the PBOMs, each participant was categorized as “at risk” or “not at risk” based on the results of each outcome measure and then categorized further as “at risk” or “not at risk” according to the recommendations for interpreting the clusters of PBOMS from the STEADI, APTA-SR and APTA-SR3.

As recommended by the STEADI, participants were categorized as “at risk” if at least 1 of the 3 PBOMs (TUG, 30SCST or 4SBT) were over the cut-off score. The recommended cut off scores are TUG >12 s, 30SCST <7–14 reps depending on age and sex, and 4SBT <10 s for any of the standing postures.

For the APTA-SR and APTA-SR3, a cut-off score of cumulative post-test probability (CpoTP) of ≥60% was considered to be ‘at risk’. CPoTP calculations were performed using the website, https://www.easycalculation.com/statistics/post-test-probability.php. A pretest probability fall prevalence rate of 30% was used for the CPoTP calculations. The recommended cutoff scores are >6.5 s for the SLST, >1.0 m/s for the SSWS, <50 points on the BBS, >11 s for the TUG, and a cutoff score of >12 s for the 5TSTS [26].

After six months, a follow up telephone interview was conducted to review the fall risk questionnaire to identify if a participant had fallen in the 6 months after the fall risk assessment.

### 2.3 Analysis

Comparative analysis of the diagnostic utilities of the different clusters of PBOMs was performed retrospectively and prospectively. Retrospective analysis compared the diagnostic abilities of the PBOMs based on any history of falls during the year preceding the fall risk assessment date. A fall event in the past year was considered to be the ‘gold standard’ for reference. Prospective analysis compared the diagnostic abilities of the clusters based on any falls that occurred within the 6-month follow-up period after the fall risk assessment date. A fall event in the 6-month period was considered to be the ‘gold standard’ for reference. The diagnostic utility for each PBOM cluster was determined by calculating the sensitivity (Sn), specificity (Sp), positive (LR+) and negative (LR-) likelihood ratios and diagnostic accuracy (Sn × prevalence + Sp × (1 − prevalence)) using the online calculator, https://www.medcalc.org/calc/diagnostic_test.php. A fall prevalence rate of 30% (pre-test probability) in community-dwelling older adults was used for the calculations.

## 3 Results

Of the 33 older adults, 51.5% (*n* = 17) reported a history of fall during the past year. 20 participants responded to the 6-month follow-up calls, of which 20% (*n* = 4) reported at least one fall. As mentioned in the assessment methodology, the fall-risk of each participant was determined separately per the referenced cut-off scores for the STEADI, APTA-SR, and APTA-SR3, which enabled the creation of 2 × 2 tables (see Appendix).

### 3.1 Retrospective analysis

The STEADI showed higher Sn than the APTA-SR (88.24% versus 82.35%, respectively) and the APTA-SR showed better Sp than the STEADI (62.50% versus 43.75%, respectively). Improved Sp resulted in a higher LR+ (2.2) and improved diagnostic accuracy, 68.46% for the APTA-SR versus 57.10% for the STEADI (Table 2). The APTA-SR3 and the STEADI demonstrated the same Sn (88.24%) and the APTA-SR3 also exhibited the same Sp (62.50%) as the APTA-SR. The APTA-SR3 had the highest LR+ (2.35) and the lowest LR- (0.19), thus providing the highest diagnostic accuracy (70.22%) of the three PBOM clusters.

**Table 2:** Diagnostic utility measures when using the gold standard of ‘History of fall’ during the past year.

	STEADI	APTA-SR	APTA-SR3
Sensitivity	88.24% (63.56–98.54)	82.35% (56.57–96.2)	88.24% (63.56–98.54)
Specificity	43.75% (19.75–70.12)	62.5% (35.43–84.8)	62.5% (35.43–84.8)
Positive Likelihood	1.57 (0.98–2.5)	2.2 (1.12–4.29)	2.35 (1.22–4.53)
Negative Likelihood	0.27 (0.07–1.11)	0.28 (0.09–0.84)	0.19 (0.05–0.73)
Accuracy	57.1% (38.76–74.11)	68.46% (50–83.45)	70.22% (51.83–84.81)

### 3.2 Prospective analysis

All of the PBOM clusters demonstrated a Sn of 100% (Table 3). However, the APTA-SR cluster demonstrated better Sp (43.75%), LR+ (1.78) and diagnostic accuracy (60.62%) than the STEADI. The APTA-SR3 cluster the same diagnostic values as the APTA-SR while only using 3 PBOMS.

**Table 3:** Diagnostic utility measures when using the gold standard of ‘fall’ during the 6-month follow-up period.

	STEADI	APTA-SR	APTA-SR3⁎
Sensitivity	100% (39.76–100)	100% (39.76–100)	100% (39.76–100)
Specificity	25% (7.27–52.38)	43.75% (19.75–70.12)	43.75% (19.75–70.12)
Positive Likelihood	1.33 (1–1.77)	1.78 (1.15–2.74)	1.78 (1.15–2.74)
Negative Likelihood	0 (0)	0 (0)	0 (0)
Accuracy	47.5% (25.1–70.66)	60.62% (36.63–81.36)	60.62% (36.63–81.36)

⁎ The values of APTA-SR-3 were the same as APTA-SR.

## 4 Discussion

The purpose of this study was to compare the diagnostic accuracies of the STEADI, APTA-SR and APTA-SR3 outcome measure clusters from two screening tools to determine fall risk in community dwelling older adults [26,43]. Considering all analyses, the APTA-SR3 demonstrated superior sensitivity, specificity, likelihood ratios and diagnostic accuracy while using the fewest outcome measures. These findings appear to suggest the APTA-SR3 cluster as a valuable and time-efficient clinical assessment tool to evaluate fall-risk.

When clustering PBOMs for fall risk assessment the goal is to maximize sensitivity without sacrificing specificity [44]. Lower sensitivity tests lead to increased probability of a false negative result by incorrectly ruling out those individuals who are actually at an elevated risk for falls. This could result in missed opportunities to provide appropriate fall risk reduction interventions such as addressing specific body structure/function impairments of an individual's balance system. On the other hand, lower specificity values can result in higher rates of false positive results. This could result in unnecessary allocation of resources for individuals who are not objectively at risk for falls resulting in overall increased healthcare costs and potentially limiting access to services for individuals who need actually them.

One drawback of using a cluster of PBOMs is the tendency for redundant assessments. When outcome measures assess the same or similar constructs this can increase alpha error (false positive error rate) by assessing the same construct or performance outcome multiple times [45]. The APTA-SR demonstrates redundancy when assessing the different balance system components discussed previously. For example, both the BBS and the SLST assess the limits of an individual's postural stability. Analyzing the same observational findings multiple times within the same group can lead to correlation among groups; this correlation falsely increases the number of statistically significant differences among the groups, and thus a higher number of false positives [46].

Conversely, the STEADI cluster appears to lack certain important constructs of balance. For example, the ability to measure limits of postural stability or the effects of sensory systems on postural control mechanisms, which are captured in BBS that is a component of the APTA-SR and APTA SR3. Mancini and Horak emphasize that each of the components of the balance system (biomechanical constraints, stability limits/verticality, anticipatory postural adjustments, postural responses, sensory orientation, stability in gait) should be assessed in PBOMs in order to accurately identify specific balance system deficits to prioritize interventions specific to the modified intrinsic risk factors related to the postural control system [47].

This study suggests the APTA-SR3 cluster could be capturing most balance-related constructs while minimizing unnecessary redundancy. The Berg Balance Scale assesses the constructs of stability limits/verticality, anticipatory postural control mechanisms and sensory integration. The TUG assesses dynamic postural control mechanisms during functional mobility (sit to stand transfer and gait) and the 5TSTS assesses the power and motor control of the lower extremity and trunk during functional sit to stand transfers. Efficient assessment of the subcomponents of the postural control system could be one reason why the diagnostic accuracy of the APTA -SR3 was superior to the APTA-SR and STEADI clusters.

Adopting a culture of measurement within clinical practice is a slow change confronted by many barriers including time constraints, difficulty for patients or clients to complete PBOMs, a lack of equipment, and a lack of healthcare provider knowledge on how to implement and use PBOMs to inform clinical decision making [15,48,49]. The use of PBOM clusters with a limited number of tests, like the APTA-SR3, can decrease the time it takes for training, implementation into standard practice, and the simplicity of the tasks within the outcome measures can account for the wide variability of physical abilities observed in the older adult population. The APTA-SR3 therefore establishes itself as a concise, clinically applicable cluster that is easy to learn and time-efficient for the busy healthcare provider to utilize to create a culture of measurement in clinical practice.

Based on the results of this study it is advisable to use the APTA-SR3 cluster as an initial fall risk screening tool. Individuals who are screened as “low risk” can be given home exercise programs centered on health and wellness principles such as the CDC guidelines for physical activity. Those who are screened as “high risk” should undergo a further comprehensive physical assessment and participate in a customized program addressing intrinsic and extrinsic modifiable risk factors.

This study has several limitations. The small sample size, especially in the prospective analysis, could decrease the statistical power of the results resulting in increased chances of type I and II errors. Additionally, reliance on self-reported fall history as the gold standard for reference may have led an underestimation of falls in the past year [50]. Additional research is warranted to determine if inclusion of self-report measures would further increase the diagnostic accuracy of the APTA-SR3 cluster in assessing fall risk.

## 5 Conclusion

The APTA-SR3 item cluster consisting of the BBS <50 points, TUG >11 s, and 5TSTS >12 s was found to have the superior diagnostic accuracy when identifying fall risk in the community dwelling adults. Clinicians should consider using this item cluster as a screening tool to identify participants who have fallen, or who are at risk for fall to properly prescribe individualized fall risk reduction strategies.

## Declaration of Competing Interest

The authors have no declaration of interest to report.

## Figures and Tables

**Appendix Table 1:** Comparison of the fall-risk screening outcomes to the gold standard of ‘History of fall’ during the past year.

		**Gold Standard**	
		Hx of Fall *n* = 17	No Hx of Fall *n* = 16
**STEADI test outcome**	At risk	15	9
	Not at risk	2	7
**APTA-SR test outcome**	At risk	14	6
	Not at risk	3	10
**APTA-SR3 test outcome**	At risk	15	6
	Not at risk	2	10

**Appendix Table 2:** Comparison of the fall-risk screening outcomes to the gold standard of ‘fall’ during 6-month follow-up.

		**Gold standard**	
		Fall n = 17	No Fall n = 16
**STEADI test outcome**	At risk	4	12
	Not at risk	0	4
**APTA-SR test outcome**	At risk	4	9
	Not at risk	0	7
**APTA-SR3 test outcome**	At risk	4	9
	Not at risk	0	7
